# Continuation of Aspirin Therapy before Cataract Surgery with Different Incisions: Safe or Not?

**DOI:** 10.1155/2018/6543937

**Published:** 2018-05-13

**Authors:** Qingjian Li, Yiwen Qian, Yu Zhang, Gaoyuan Sun, Xian Zhou, Zhiliang Wang

**Affiliations:** Department of Ophthalmology, Huashan Hospital, Fudan University, Shanghai, China

## Abstract

**Purpose:**

To assess whether to continue aspirin therapy while having uncomplicated phacoemulsification cataract surgery with different incisions.

**Methods:**

Consecutive patients having cataract surgery under topical anesthesia with different incisions between May 2016 and August 2017 were followed. 236 eyes of 166 patients on routine aspirin therapy were randomized into 2 groups: continuation group, 112 eyes; discontinuation group, 124 eyes. 121 eyes of 94 patients on no routine anticoagulant therapy were used as the control group. Patients were examined 1 day preoperatively and 1 day and 7 days postoperatively. Intraoperative and postoperative complications were recorded.

**Results:**

Statistically, there was no significant difference about postoperative BCVA among three groups. A higher incidence of subconjunctival hemorrhage was shown in the continuation group than in the discontinuation group and the control group (17.0% versus 8.1%, *p*=0.038; 17.0% versus 7.4%, *p*=0.025, resp.). Although corneal edema was greater in clear corneal incision cases than that of scleral tunnel incision cases (22.5% versus 12.0%, *p*=0.009), subconjunctival hemorrhage was greater in scleral tunnel incision cases (14.9% versus 6.6%, *p*=0.011). Subgroup analyses revealed that patients of scleral tunnel incision who continued taking aspirin had a higher incidence of subconjunctival hemorrhage compared with those who discontinued (25.5% versus 10.9%, *p*=0.038), but no same conclusion in clear corneal incision cases (8.8% versus 5.0%, *p*=0.483).

**Conclusions:**

The outcomes indicated that phacoemulsification cataract surgery under topical anesthesia could be safely performed without ceasing systemic aspirin therapy. Clear corneal incision could be a better choice in patients treated with aspirin.

## 1. Introduction

Aspirin has been the cornerstone of preventing thromboembolic complications in patients with cerebrovascular, coronary artery, and peripheral vascular diseases. It is a weak anticoagulant and acts chiefly through the passivation of platelet cyclooxygenase 1 and suppression of thromboxane A2 production. This regimen has significantly reduced risks for morbidity and mortality due to vascular events. The potential benefit of aspirin and the low rate of adverse effects have made aspirin intaking fashionable in the elderly population [1–5]. Cataract surgery is one of the most common surgeries performed in elderly patients [6]. It is estimated that more than 20% of patients routinely took aspirin before cataract surgery [7, 8]. Patients receiving long-term antiplatelet therapy face a significant clinical challenge when they need a cataract surgery. If antiplatelet therapy is suspended, there is a risk for vascular events; however, continuation of aspirin treatment may be closely associated with serious perioperative bleeding complications [9, 10]. Hence, there is an issue of whether the risk of thromboembolic events associated with temporarily ceasing antiplatelet treatment before surgery outweighs the advantages of fewer hemorrhagic events. However, at present, there is no clear consistent recommendation in these cases before cataract surgery.

This prospective randomized study was undertaken to compare the incidence of intraoperative and postoperative complications in patients continuing aspirin therapy and those ceasing it while having phacoemulsification cataract surgery with different incisions.

## 2. Methods

### 2.1. Patients

This prospective randomized study included consecutive patients who had phacoemulsification cataract surgery under topical anesthesia with different incisions between May 2016 and August 2017 at Huashan Hospital, Fudan University, Shanghai, China. Two hundred thirty-six eyes of 166 patients on routine aspirin therapy were randomized into 2 groups: the continuation group, aspirin continued up to the time of surgery, 112 eyes; the discontinuation group, aspirin discontinued for 3 to 7 days before surgery till 1 day after surgery, 124 eyes. One hundred twenty-one eyes of 94 patients on no routine anticoagulant therapy were used as the control group. The study protocols were accepted by the Institutional Review Board of Huashan Hospital affiliated to Fudan University and performed in accordance with the tenets of the Declaration of Helsinki. Written informed consent was gained from each participant.

### 2.2. Exclusion Criteria

Ocular exclusion criteria were previous operation in the same eye, having phacoemulsification cataract surgery combined with other surgeries (e.g., trabeculectomy or pars plana vitrectomy), pupil dilating to be short of 4.0 mm, nuclear hardness over grade IV, uncontrolled glaucoma, floppy iris syndrome, and existence of pathological vessels in the operative eye (e.g., iris neovascularization or proliferative diabetic retinopathy). Systemic exclusion criteria were severe renal failure, fasting glucose > 9 mmol/L, and blood pressure > 170/90 mmHg. The patients on other kinds of antiplatelets and/or anticoagulants were excluded.

### 2.3. Data Collection

All eligible patients had a complete preoperative evaluation including ocular and systemic examination. The ophthalmologic evaluation included slit-lamp biomicroscopy, best-corrected visual acuity (BCVA), intraocular pressure (IOP), and funduscopic examination. The BCVA was converted to the logMAR value, which was used in all statistical analyses. IOP elevation and hypotony were defined as IOPs over 30 mmHg and less than 9 mmHg, respectively [11, 12]. The systemic evaluation included blood, electrocardiogram, and blood pressure. After surgery, the patients were followed up at 1 day and 7 days. All intraoperative and postoperative complications were recorded. Special concern was given to evidence of hemorrhagic complications.

### 2.4. Surgical Technique

Three hundred fifty-seven cases of phacoemulsification cataract surgery through different incisions were performed by 1 of 8 skilled surgeons at the same institution. Compound tropicamide eye drop which contained tropicamide (0.5%) and phenylephrine hydrochloride (0.5%) was used as preoperative mydriatic regularly. Topical anesthesia of oxybuprocaine hydrochloride (20 ml: 80 mg) was used 3 times before cataract surgery. Different incisions were applied according to surgeon's preference. Five surgeons performed through clear corneal incision, while three surgeons preferred scleral tunnel incision. In the scleral tunnel incision cases, after a conjunctival incision at 10:30, cauterization was applied to coagulate conjunctival and episcleral vessels. All surgeons did not know whether the patient was taking aspirin up to the time of surgery or had discontinued the medication some days earlier.

### 2.5. Statistical Analysis

SPSS for Windows Version 19.0 (SPSS, Inc., Chicago, IL, USA) was applied for all statistical analysis. Random number table was used for randomization. A two-tailed *p* value < 0.05 was considered statistically significant. The Kolmogorov–Smirnov test was applied to analyze the distribution of continuous variables. Continuous variables were expressed as mean ± standard deviation (SD) for normally distributed data and median (interquartile range (IQR)) for nonnormally distributed data. Categorical variables were expressed as absolute numbers and percentages. Student's *t*-test was applied for normally distributed data, and the Wilcoxon rank sum test was applied for nonnormally distributed data. The chi-square test was used to compare categorical variables between the continuation group and discontinuation group. When small numbers revealed that the chi-square test might be invalid, the Fisher exact test was applied.

## 3. Results

Phacoemulsification cataract surgery under topical anesthesia was performed in 357 eyes of 260 patients. One hundred sixty-six patients took aspirin preoperatively on a daily basis.  Table 1 characterizes demographic features. The dosage of aspirin intake was 100 mg/day routinely.  Table 2 characterizes systemic indication for antiplatelet therapy. The most common systemic clinical indication for aspirin therapy was thromboembolic disease (cerebral infarction, myocardial infarction, venous thrombosis, etc.), which accounted for 70.3% of patients in our study. Other less common indications, which accounted for an additional 10.6%, included atrial fibrillation, mechanical mitral, coronary artery bypass graft, cardiac pacemaker, and dilated cardiomyopathy. But 19.1% of the patients presented no certain systemic clinical indications for aspirin therapy on a daily basis. The three groups were equally distributed in sex, age, BCVA, IOP, types of incision, surgeons, and the proportions of patients having surgery to both eyes. Statistical analysis showed no significant differences between the continuation group and discontinuation group in duration of aspirin intake and systemic indication for antiplatelet therapy.

Table 3 characterizes the postoperative BCVA and IOP in three groups, and there was no statistic difference among them. One day postoperatively, BCVA was improved from 0.58 ± 0.19 to 0.42 ± 0.39 (in the continuation group), 0.61 ±0.18 to 0.45 ± 0.39 (in the discontinuation group), and 0.62 ±0.17 to 0.47 ± 0.45 (in the control group), respectively. And one week postoperatively, BCVA was improved to 0.24 ± 0.19 (in the continuation group), 0.27 ± 0.21 (in the discontinuation group), and 0.27 ± 0.20 (in the control group), respectively.

Table 4 characterizes the hemorrhagic and nonhemorrhagic complications noted in the three groups. None of the patients suffered from thromboembolic events in our study during the follow-up period. No surgery was canceled or postponed because of bleeding complications. Subconjunctival hemorrhage was the most frequent hemorrhagic complication. There was a higher incidence of subconjunctival hemorrhage in the continuation group than in the discontinuation group and the control group (17.0% versus 8.1%, *p*=0.038; 17.0% versus 7.4%, *p*=0.025, resp.).

The incidence of subconjunctival hemorrhage was significantly greater in the scleral tunnel incision cases than in the clear corneal incision cases (14.9% versus 6.6%, *p*=0.011). Tables 5 and 6 characterize the hemorrhagic and nonhemorrhagic complications in different incision cases. Subgroup analyses revealed that patients of scleral tunnel incision who continued taking aspirin had a higher incidence of subconjunctival hemorrhage compared with those who stopped treatment before surgery (25.5% versus 10.9%, *p*=0.038), but there was no same conclusion in the clear corneal incision cases (8.8% versus 5.0%, *p*=0.483, Fisher's exact test). No patient requested additional clinic visits because of the subconjunctival hemorrhage.

Corneal edema was the most frequent nonhemorrhagic complication. We found no statistical difference of nonhemorrhagic complication, such as corneal edema, elevated IOP, hypotony, and posterior capsule rupture among the continuation group, discontinuation group, and control group. The incidence of corneal edema was significantly greater in the clear corneal incision cases than in the scleral tunnel incision cases (22.5% versus 12.0%, *p*=0.009), but this complication faded away spontaneously within a week without clinical consequences.

Another common postoperative nonhemorrhage complication was elevated IOP or hypotony. We found no statistic difference of elevated IOP or hypotony among the continuation group, discontinuation group, and control group. And 78.9% (15/19) of disordered IOP returned to normal within 7 days.

In the continuation group, one patient had an intraoperative posterior capsule rupture and received secondary implantation of intraocular lens (IOL) without clinical consequences after one month. In the discontinuation group, a patient who had an intraoperative rupture of posterior capsule had instant implantation of IOL and maintained good visual acuity. There were no cases of choroidal/suprachoroidal hemorrhage, vitreous hemorrhage, distorted pupil, retinal detachment, or endophthalmitis.

## 4. Discussion

Antiplatelet therapy is continually encountered in patients desiring cataract surgery. Because of the progressive aging of the population, aspirin users are more likely to be elderly and, therefore, more likely to desire a surgical procedure. The clinical challenge is to conclude whether to continue or to interrupt antithrombotic therapy. It is considered that 10% of all patients receiving oral antiplatelet medications request interrupted treatment for surgery or an invasive procedure each year [10]. In the past studies, 25.6% of the Canadian Society of Cataract and Refractive Surgery members discontinued aspirin routinely before cataract surgery [13]. 22.5% of routine aspirin users ceased the medication during the perioperative period of cataract surgery [8]. To reduce the risk of intraoperative and postoperative hemorrhagic complications, the ophthalmologists preferred to ceasing aspirin 3 to 7 days prior to cataract surgery and interrupt use 1 to 2 days postoperatively, despite the potential risk of thromboembolic events [13–15].

For decades, whether to stop antiplatelet therapy before cataract surgery remains no decision. Recent studies showed the safety of antiplatelet medications in cataract surgery and no increase in sight-threatening complications, such as severe hemorrhage affecting visual acuity or retinal detachment. [8, 16, 17]. The American College of Chest Physicians and American Academy of Neurology advised continuous use of antiplatelets before cataract surgery [18, 19]. The cataract in the Adult Eye Preferred Practice Pattern of the American Academy of Ophthalmology gave a 1- grade recommendation that aspirin should be discontinued perioperatively only if the risk of bleeding outweighed its potential benefit [20]. Same as these clinical researches, in our experience, there was no statistic difference in postoperative BCVA among the three groups. What's more, except one case of hyphema postoperatively in the continuation group, no case of choroidal/suprachoroidal hemorrhage, vitreous hemorrhage, distorted pupil, retinal detachment, or endophthalmitis happened in patients with aspirin treatment.

We observed a higher incidence of subconjunctival hemorrhage in patients who continued aspirin therapy compared with those who stopped treatment. This complication was self-limiting without permanent sequelae. Cauterization was applied to coagulate conjunctival and episcleral vessels in the scleral incision cases, but the incidence of subconjunctival hemorrhage was significantly greater in the scleral tunnel incision cases than in the clear corneal incision cases. In 2006, the study by Kumar et al. [21] showed no difference in incidence of subconjunctival hemorrhage between the aspirin group and control group. But the primary diseases between the two groups were unbalanced and it was relatively a small sample study.

Results of the subgroup analyses showed that patients of scleral tunnel incision who continued taking aspirin had a higher incidence of subconjunctival hemorrhage compared with those who discontinued for 3 to 7 days before surgery, but there is no same conclusion in the clear corneal incision cases. This meant that aspirin would markedly increase the risk of subconjunctival hemorrhage in scleral tunnel incision cases. No patient requested additional clinic visits because of the subconjunctival hemorrhage. In a study by Kobayashi [14], patients on antiplatelets and/or anticoagulants continuously showed a higher incidence of subconjunctival hemorrhage compared with the discontinuation group (16.5% versus 10.8%, *p*=0.0309). And the possible reason for bleeding might be the use of subtenon's anesthesia and it was out of date.

One patient who continued taking aspirin in the clear corneal incision cases had a postoperative hyphema (<2.0 mm) that resolved spontaneously in two weeks without affecting visual acuity. The possible explanation was injury of iris vessels during phacoemulsification. The happening of bleeding complication was probably coincidental.

Corneal edema was the main nonhemorrhagic complication. In our study, there was no significant difference in nonhemorrhagic complications among the continuation group, discontinuation group, and control group. The incidence of corneal edema was significantly greater in the clear corneal incision cases than in the scleral tunnel incision cases, but this complication faded away spontaneously within a week without clinical consequences. The possible explanation for the nonhemorrhagic complication is the excessive manipulation through the incision.

The major advantage of this study was comparisons of the incidence of intraoperative and postoperative complications among the three groups in different incision cases. The patients of scleral tunnel incision who continued taking aspirin had a higher incidence of subconjunctival hemorrhage compared with those who stopped treatment before surgery, but there was no same conclusion in the clear corneal incision cases. The patients with clear corneal incision had more chance of corneal edema but less subconjunctival hemorrhage compared to patients with scleral tunnel incision. Subconjunctival hemorrhage usually lasts for 2-3 weeks and longer in patients with aspirin treatment. But corneal edema fades away spontaneously within a week without clinical consequences. So, we hold the opinion that clear corneal incision is better than scleral tunnel incision in patients treated with aspirin.

The patients on other kinds of antiplatelets and/or anticoagulants were excluded from this study. Therefore, we believe that the results represent a true reflection of the hemorrhagic risks associated with the continuation of aspirin uncomplicated by the effect of other medicines.

This study has limitations. First, because the number of patients was relatively small, the power of analysis was not great enough to discover tiny differences that might have been existent. Second, there were certain indications for use of aspirin that put most routine users at higher risk for bleeding complications than those not on the medication. For example, there was no operative history of percutaneous coronary intervention in the control group. A comparison between the continuation group and discontinuation group is likely to reduce the bias.

Although patients taking aspirin may be at higher risk for bleeding complications, the practical situation that lots of patients are advised to stop or alter antiplatelet therapy before routine cataract surgery may increase risk of vascular event. If a vascular event did occur, it was much more fearful and resulted in a higher mortality. Furthermore, the technique placing incisions in the avascular clear cornea under topical anesthesia reduces the risk of bleeding complications. A clear consistent recommendation to maintain antiplatelet therapy during phacoemulsification cataract surgery should be instituted by national and international ophthalmic societies.

## 5. Conclusions

The outcomes in our study indicate that phacoemulsification cataract surgery under topical anesthesia could be safely performed without ceasing systemic aspirin therapy. Clear corneal incision could be a better choice in patients treated with aspirin compared with scleral tunnel incision.

## Figures and Tables

**Table 1:** Demographic characteristics.

Parameter	Continuation group	Discontinuation group	Control group	*p* value
Patients, *n*	81	85	94	—

Eye, *n*	112	124	121	—
Sex, *n*				0.223^a^
Male	46	39	42	
Female	35	46	52	

Age, year				0.193^b^
Mean ± SD	75.2 ± 8.6	76.3 ± 7.3	74.5 ± 8.0	
Range	50–93	55–90	58–89	

BCVA, logMAR				0.165^b^
Mean ± SD	0.58 ± 0.19	0.61 ± 0.18	0.62 ± 0.17	

IOP, mmHg				0.210^b^
Mean ± SD	15.9 ± 2.6	15.2 ± 2.6	15.6 ± 2.9	

Aspirin duration, month				0.119^c^
Median (IQR)	60 (24–96)	60 (36–120)	—	

Type of incision, *n*				0.706^a^
Corneal incision	57	60	65	
Scleral incision	55	64	56	

BCVA = best-corrected visual acuity; IOP = intraocular pressure; ^a^chi-square test; ^b^Student's *t*-test; ^C^Wilcoxon rank sum test.

**Table 2:** Systemic indication for antiplatelet therapy.

Systemic indication for antiplatelet therapy	Eyes, *n* (%)		*p* value
	Continuation group (*n*=112)	Discontinuation group (*n*=124)	
Thromboembolic disease	84 (75.0)	82 (66.1)	0.136^a^
Atrial fibrillation	4 (3.6)	7 (5.6)	0.450^a^
Mechanical mitral	1 (0.9)	4 (3.2)	0.373^d^
Coronary artery bypass graft	1 (0.9)	3 (2.4)	0.624^d^
Cardiac pacemaker	1 (0.9)	2 (1.6)	1.000^d^
Dilated cardiomyopathy	1 (0.9)	1 (0.9)	1.000^d^
Precaution	20 (17.8)	25 (20.2)	0.653^a^

Precaution = patients without certain systemic clinical indication; ^a^chi-square test; ^d^Fisher's exact test.

**Table 3:** Postoperative BCVA and IOP by group.

Parameter	Continuation group (*n*=112)	Discontinuation group (*n*=124)	Control group (*n*=121)	*p* value
One day postoperative				
BCVA, logMAR	0.42 ± 0.39	0.45 ± 0.39	0.47 ± 0.45	0.698^b^
IOP, mmHg	17.5 ± 4.5	18.0 ± 5.0	17.6 ± 4.9	0.729^b^

One week postoperative				
BCVA, logMAR	0.24 ± 0.19	0.28 ± 0.21	0.27 ± 0.20	0.425^b^
IOP, mmHg	16.2 ± 2.8	16.1 ± 3.0	15.8 ± 3.0	0.531^b^

^b^Student's *t*-test.

**Table 4:** Incidence of complications by group.

Complication	Eyes, *n* (%)			*p* value
	Continuation group (*n*=112)	Discontinuation group (*n*=124)	Control group (*n*=121)	
Hemorrhagic				
Subconjunctival hemorrhagic	19 (17.0)	10 (8.1)	9 (7.4)	0.032^a^^∗^
Hyphema	1 (0.9)	0 (0)	0 (0)	0.334^d^

Nonhemorrhagic				
Corneal edema	21 (18.8)	22 (17.7)	19 (15.7)	0.821^a^
Posterior capsule rupture	1 (0.9)	1 (0.8)	2 (1.7)	0.790^d^
Hypotony (<9 mmHg)	1 (0.9)	3 (2.4)	4 (3.3)	0.455^d^
IOP elevation (>30 mmHg)	3 (2.7)	6 (4.8)	2 (1.7)	0.338^d^

^∗^ *p* < 0.05; ^a^chi-square test; ^d^Fisher's exact test.

**Table 5:** Incidence of complications in scleral tunnel incision cases.

Complication	Eyes, *n* (%)			*p* value
	Continuation group (*n*=55)	Discontinuation group (*n*=64)	Control group (*n*=56)	
Hemorrhagic				
Subconjunctival hemorrhagic	14 (25.5)	7 (10.9)	5 (8.9)	0.027^a^^∗^
Hyphema	0 (0)	0 (0)	0 (0)	—

Nonhemorrhagic				
Corneal edema	7 (12.7)	8 (12.5)	6 (10.7)	0.937^a^
Posterior capsule rupture	0 (0)	1 (1.6)	0 (0)	0.418^d^
Hypotony (<9 mmHg)	0 (0)	2 (3.1)	2 (3.6)	0.386^d^
IOP elevation (>30 mmHg)	1 (1.8)	4 (6.2)	0 (0)	0.105^d^

^∗^ *p* < 0.05; ^a^chi-square test; ^d^Fisher's exact test.

**Table 6:** Incidence of complications in clear corneal incision cases.

Complication	Eyes, *n* (%)			*p* value
	Continuation group (*n*=57)	Discontinuation group (*n*=60)	Control group (*n*=65)	
Hemorrhagic				
Subconjunctival hemorrhagic	5 (8.8)	3 (5.0)	4 (6.2)	0.702^d^
Hyphema	1 (1.8)	0 (0)	0 (0)	0.332^d^

Nonhemorrhagic				
Corneal edema	14 (24.6)	14 (23.3)	13 (20.0)	0.821^a^
Posterior capsule rupture	1 (1.8)	0 (0)	2 (3.1)	0.401^d^
Hypotony (<9 mmHg)	1 (1.8)	1 (1.7)	2 (3.1)	0.833^d^
IOP elevation (>30 mmHg)	2 (3.5)	2 (3.3)	2 (3.1)	0.991^d^

^a^Chi-square test; ^d^Fisher's exact test.

## Data Availability

The data sets used and/or analyzed during the current study are available from the corresponding author on reasonable request.
